# Huxie Huaji Ointment Induced Apoptosis of Liver Cancer Cells In Vivo and In Vitro by Activating the Mitochondrial Pathway

**DOI:** 10.1155/2021/9922059

**Published:** 2021-07-15

**Authors:** Yuan Cai, Qing Du, Tian-Hao Deng, Bing-Bing Shen, Yan-Mei Peng, Pu-Hua Zeng, Song-Ren Yu

**Affiliations:** ^1^Institute of Traditional Chinese Medicine, Hunan Academy of Chinese Medicine, Changsha 410006, China; ^2^Jiangxi University of Chinese Medicine, Nanchang, Jiangxi 330006, China

## Abstract

Huxie Huaji (HXHJ) Ointment is a famous traditional Chinese medicinal prescription and is commonly used for the clinical treatment of hepatocellular carcinoma by boosting immunity and detoxification. However, the scientific evidence for the effect of HXHJ Ointment on hepatocellular carcinoma and the underlying molecular mechanism are lacking. The present study aimed to identify the effects of HXHJ Ointment on hepatocellular carcinoma in vitro and in vivo as well as investigating the mechanistic basis for the anticancer effect of HXHJ ointment. First, liquid chromatography-mass spectrometry was used to verify the composition of HXHJ Ointment and quality control. Second, in vitro, Cell Counting Kit (CCK8) cell viability assay and Hoechst 33342 staining assay were performed to explain the cell apoptosis. The protein levels of tumor suppressor protein (*p*53), B-cell lymphoma 2 gene (Bcl-2), cytochrome C (Cyt-C), and aspartate proteolytic enzyme-3 (caspase-3) were examined by immunofluorescence. Finally, in vivo, hematoxylin and eosin (H&E) staining was used to observe the pathological changes in hepatocellular carcinoma samples. Western blots and immunohistochemistry were used to detect the anticancer properties of HXHJ ointment. The results in vitro showed that 20% HXHJ Ointment serum could significantly inhibit HepG2 cell proliferation, increased tumor suppressor gene *p*53, downregulated antiapoptotic protein Bcl-2, promoted the release of mitochondrial Cyt-C, activated caspase-3, and induced HepG2 cell apoptosis. Furthermore, in vivo experiments showed that HXHJ Ointment could effectively inhibit tumor growth in nude mice xenotransplanted with HepG2 cells, changed the morphology of tumor cells, and regulated the expression of apoptosis-related protein pathway *p*53/Bcl-2/Cyt-C/caspase-3. HXHJ Ointment can significantly inhibit the development of hepatocellular carcinoma, and its mechanism may be related to the regulation of *p*53/Bcl-2/Cyt-C/caspase-3 signaling pathway to induce cell mitochondrial apoptosis.

## 1. Introduction

Hepatocellular carcinoma (HCC) is a more common malignancy than the majority of cancers and ranks second in the world's top causes of cancer-related mortality [1]. Progress has been made in the diagnosis and therapy and we are encouraged by the recent success, while survival benefits remain modest due to the difficulty in being diagnosed early and the disease progressing quickly [2]. Currently, the major clinical treatment strategies for HCC include surgery, radiotherapy, and chemotherapy [3]. Unfortunately, radiotherapy and chemotherapy are not suitable for everyone and have the drawbacks of “kill a thousand enemies, hurt yourself eight hundred.” Moreover, those who have undergone hepatectomy or liver transplantation are frequently subject to survival declines due to recurrence and metastasis [4]. Thus, there is an urgent need for more effective treatment to reduce the rate of recurrence and metastasis, relieve clinical symptoms of HCC, and improve long-term patient survival and high quality of life.

Studies on looking for the new drugs that can kill tumor cells and enhance the body's immunity may provide insight into novel therapeutic strategies. Traditional Chinese medicine (TCM), especially the formulas, has been used clinically for more than five thousand years in China and Asia. Various studies have shown that traditional Chinese medicine formulas (TCMF) have a marked effect on the treatment of liver cancer [5]. With an overall concept, it has a unique therapeutic effect and fewer side effects on HCC patients. It can not only inhibit tumor growth but also alleviate patients' clinical symptoms and improve their quality of life [6].

Apoptosis is one such cell death pathway which allows a damaged or stressed cell to deconstruct in a regulated manner without causing inflammation and has garnered much attention in the cancer field, with hopes of eliminating malignant cells through its activation. Accumulating evidence has suggested that the mitochondrial pathway of apoptosis in a cell is one of the most important hallmarks of HCC [7, 8]. In mammals, activation of caspases is under the tight control of the Bcl-2 family proteins, named in reference to the first discovered mammalian cell death regulator [9, 10]. These proteins mainly act by regulating the release of caspases activators from mitochondria. Mitochondria appear today as the central executioner of apoptosis. Cyt-C is one of the apoptotic factors released by mitochondria into the cytoplasm and can bind to Apaf-1 (apoptosis protease activating factor) and induce cell apoptosis. The *p*53 tumor suppressor protein plays a central role in the regulation of apoptosis. Thus, documentation of changes to the expression of these markers would provide insights as to the mechanism whereby TCMF might be of value in the treatment of HCC.

Huxie Huaji (HXHJ) Ointment is a classic anticancer prescription and clinical studies have shown that HXHJ Ointment can improve patient quality of life and extend survival time when used to treat advanced primary liver cancer. It can significantly enhance the therapeutic effect and reduce tumor resistance in combination with Western Medicine chemotherapy. In our previous study, we found that HXHJ Ointment could significantly inhibit the proliferation of H_22_ cells by inducing the imbalance of the expression ratio of Bax and Bcl-2 proteins *in vitro* [11]. In the current study, our aim was to confirm the effects of HXHJ Ointment on HepG2 cells and tumor growth. Elucidation of the apoptosis mechanisms and molecular biology in HCC underlying HXHJ Ointment will provide more comprehensive and precise insight into clinical guidance.

## 2. Materials and Methods

### 2.1. Materials

Huxie Huaji (HXHJ) Ointment was made up of ten herbs, *Codonopsis Radix*, *Astragali Radix*, *Herba Hedyotidis Diffusae*, *Agrimonia pilosa Ledeb.*, *Scutellaria barbata* D. Don., *Reynoutria japonica* Houtt., *Smilax china* L., *Fructus Ligustri Lucidi*, *Buthus martensii Karsch*, and *Gekko swinhonis Guenther* in the proportion 20 : 30 : 15 : 15 : 10 : 10 : 5 : 3 : 2 : 2. All the crude drugs were obtained from the Affiliated Hospital of Hunan Academy of Traditional Chinese Medicine (Changsha, China). The voucher specimens were stored in the Herbal Medicine Room (Institute of Traditional Chinese Medicine, Hunan Academy of Traditional Chinese Medicine, Changsha). Cisplatin injection was purchased from Baxter International Co., Ltd. (Illinois, USA), with batch number 8H259A.

The CCK8 kit was purchased from Shanghai Qihai Biomedical Technology Co., Ltd. (Shanghai, China). DMEM/F12 medium and fetal bovine serum (FBS) were purchased from Gibco (New York, USA). Nuclear dye Hoechst 33258 and DAPI were purchased from Solarbio (Beijing, China). Bcl-2, caspase-3, Cyt-C, and *p*53 primary antibodies were purchased from Proteintech (Wuhan, China). GAPDH was purchased from ImmunoWay (New York, USA). Phosphodiesterase inhibitor, goat anti-mouse secondary antibody, and goat anti-rabbit secondary antibody were purchased from Auragene (Changsha, China). DAB color reagent kit was purchased from Beijing Zhongshan Jinqiao Biotechnology Co., Ltd. (Beijing, China).

### 2.2. HXHJ Sample Preparation and Quality Control by Using HPLC and HPLC-Q-TOF

#### 2.2.1. HXHJ Sample Preparation

**Figure 1:**
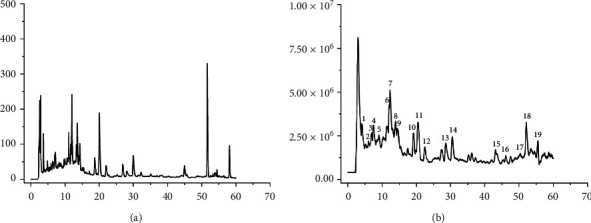
Representative HPLC chromatograms (a) and mass spectrum ESI (-) chromatograms (b) of HXHJ.

According to the Chinese Pharmacopoeia (2015 edition), the HXHJ sample mixture was immersed in deionized water (6 times its total weight) overnight. Subsequently, they were decocted for 1 hour for the first time. Then, the extraction was repeated by adding deionized water (4 times its weight) and boiled for 1 hour. Finally, the two extracts were mixed. A part of the extract was concentrated and centrifuged to an appropriate concentration for HPLC-MS analysis, and the remaining part was freeze-dried for further use. The final amount of the extracted HXHJ powder was 230.34 g (yield, 14.28% (w/w)). The main pharmacologically active ingredients are known (Figure 1).

#### 2.2.2. HPLC Conditions

The chromatography analytical procedures were performed on an Agilent 1260 HPLC system (Agilent Technologies, Santa Clara, CA, USA). Chromatographic separation was performed on an Agilent Eclipse XDB-C18 column (4.6 × 250 mm, 5 *μ*m). The mobile phase consisted of A (0.1% formic acid in water) and B (Acetonitrile) was programmed as follows: 0–5 min, 5–10% B; 5–25 min, 10–45% B; 25–40 min, 45–65% B; and 40–65 min, 65–100% B. The flow rate was 1 mL/min while the column temperature was set at 30°C. Spectra were recorded from 190 to 400 nm, while the chromatogram was acquired at 330 nm.

#### 2.2.3. Mass Spectrometry Conditions

Agilent 6530 Accurate-Mass Q-TOF LC/MS system (Agilent Technologies, Santa Clara, CA) was equipped with an electrospray ionization (ESI); MS data were acquired across the range m/z 100–1500 in positive and negative ion modes. The operating conditions were as follows: nitrogen as dry gas with temperature at 325°C and flow rate at 5.0 L/min, and sheath gas with temperature at 400°C and flow rate at 12 L/min; pressure of nebulizer, 55 psi; capillary voltage, 3500 V; skimmer, 65 V; OCT 1 RF Vpp, 750 V; and fragmentor voltage, 130 V. The mass axis was calibrated using the mixture. For full scan MS analysis, the spectra were recorded in the range of m/z 70–3200. All operations, acquisition, and analysis of data were monitored by Agilent HPLC-ESI-Q-TOF-MS MassHunter Acquisition Software version A.01.00 and operated under MassHunter Acquisition Software Version B.05.00.

### 2.3. Preparation of HXHJ-Medicated Serum

Adult Sprague Dawley rats (180–200 g) were purchased from Hunan Slack Jingda Experimental Animal Co., Ltd. The rats were kept in cages under the conditions of a light/dark cycle of 12 h/12 h (lighting time 7 : 00–19 : 00), and they have free access to feed and water. This study was approved and conducted by the animal ethics committee of Hunan Academy of Chinese Medicine (approval number: 20200025). All procedures were performed in accordance with the Guidance Suggestions for the Care and Use of Laboratory Animals, formulated by the Ministry of Science and Technology of China.

20 rats were randomly divided into HXHJ group (*n* = 10) and control group (*n* = 10). The HXHJ group was treated by intragastric administration of HXHJ (2.44 g/kg) twice a day for 3 days. One hour following the final administration, the blood sample was collected from the abdominal aorta and centrifuged (3000 ×g, 15 min). The HXHJ-medicated serum was inactivated by heating (56°C, 30 min), filtered through a 0.22 *μ*m filter, and stored at −80°C. Rats in the control group took purified water orally with the same protocol to prepare blank serum.

### 2.4. Cell Culture and Treatment

Human hepatocellular carcinoma (HepG2) cells were obtained from the China Center for Type Culture Collection (CCTCC, Wuhan, China). The cells were grown in sterile plastic flasks or plates in DMEM medium supplemented with 10% fetal bovine serum (FBS), 10 *μ*l/mL penicillin-streptomycin solution, and incubated at 37°C, 5% CO_2_ in a humidified atmosphere. The cells were then treated separately with serum containing HXHJ of different solubility and cisplatin (positive control). Cells were collected at different time points according to experiment requirements.

### 2.5. Cell Counting Kit

The effect of serum containing HXHJ on the proliferation of HepG2 cells was evaluated by CCK-8 assay. Cells were seeded into 96-well culture plates at a density of 1 × 10^3^ cells/well and incubated in a complete medium overnight. Cells were then cultured in 100 *μ*L of fresh medium containing various concentrations of the medicated serum (5%, 10%, 15%, and 20%) and cisplatin (12.5, 25, 50, and 100 *μ*mol/L) for 24 h. Next, 20 *μ*L CCK-8 stock solution was added to each well for 4 h. Finally, a multifunctional microplate reader (Thermo, USA) was used to measure the OD value of each well at 450 nm wavelength and calculate the cell inhibition rate of each group.

### 2.6. HepG2 Cell Morphology Observation

The cells were seeded into a 24-well culture plate at a density of 2 × 10^4^ cells/well and incubated overnight in a complete medium. The cells were randomly divided into a normal control group, 20% blank serum group, 100 *μ*mol/L cisplatin group, and 20% HXHJ drug-containing serum group. The morphological changes of each group were observed on an inverted microscope (OLYMPUS, Japan).

### 2.7. Hoechst Staining

To examine cell apoptosis, the Hoechst 33342 staining assay was performed as previously reported [12] with minor revision. HepG2 cells were seeded into 6-well plates for 12 h and then treated with different concentrations of PE for 24 h. Subsequently, the culture medium was removed and the HepG2 cells were stained with Hoechst 33342 (final concentration 1.0 mg/mL) for 20 min, the apoptotic cell changes in morphology such as chromatin condensation and nuclear shrinking could be characterized. The fluorescence was observed under a fluorescence microscope and images were captured with an electronic camera (Olympus, Tokyo; 100 magnification).

### 2.8. TUNEL Staining

Take the logarithmic growth phase cells, inoculate them in a black 96-well culture plate (1 × 10^5^ per well), and culture them for 24 h. First, HepG2 cells were fixed with 4% cell fixative solution for 45 min, then 0.25% Triton‐100 was added dropwise for 15 min and then treated with 3% H_2_O_2_ for 2 min. Second, the preprepared tunel reaction mixture (50 *μ*l TdT + 450 *μ*l fluorescein-labeled dUTP) was added and incubated for 1 h in a humid chamber at 37°C in the dark. Third, DAPI working solution (100 g/L) was added to each well and incubated at room temperature for 15 min in the dark. Finally, the cell apoptosis under a laser confocal microscope was observed (Olympus, Tokyo; 100 magnification). Apoptosis rate/% = number of apoptotic cells/total number of cells × 100%, and the average value was used as an index of apoptosis.

### 2.9. Immunofluorescence Analysis

HepG2 cells were inoculated into PE black 96-well cell culture plate and fixed with 4% paraformaldehyde for 45 min, 0.25% Triton-100 for 15 min, and 5% BSA for 10 min. Rabbit anti-human Cyt-C, *p*53, Bcl-2, caspase-3, and primary antibody dilution (1 : 100) were added to the cell culture plate and incubated at 4°C overnight. FITC-labeled goat anti-rabbit secondary antibody dilution (1 : 400) was added, the liquid was discarded, the DAPI diluent (1 : 800) was added and incubated in dark at room temperature for 15 min. Protein fluorescence intensity was analyzed and processed using the OPERETTA type HCA (PerkinElmer, Massachusetts, USA) and each group of the randomly selected five horizons. The first DAPI labeled nuclei were circled, and FITC-labeled positive expression cells were found. The total fluorescence intensity value of the positive cells was calculated.

### 2.10. Establishment of Mouse Tumor Model

This research was reviewed and approved by the Experimental Animal Welfare Ethics Committee of Hunan An-sheng-mei Animal Research Institute (approval number: ASM2019043). The design and plan fully considered the principles of safety and fairness. Four-week-old female BALB/c nu/nu mice (15–20 g) were maintained in a specific pathogen-free environment. After 2 weeks of adaptation, the mice were injected subcutaneously in the right armpit with 0.2 mL HepG2 cells (2 × 10^7^/mL) and then randomized into treatment groups as follows: model group (saline, *n* = 6), cisplatin group (4 mg/kg, *n* = 6), and HXHJ-treated group (1 mg/kg, *n* = 6). The mice in the model control (normal saline) and HXHJ-treated group received intragastric administration (i.g.) every day. The mice in the positive groups receive an intraperitoneal injection (i.p.) every three days.

### 2.11. Tumor Volume and Weight Determination

Body weights and tumor volumes were measured every two days with daily general observations. Tumor growth was monitored by a digital Vernier, *V* = (*a*×*b*^2^)/2 (cm^3^) (*a*: tumor long diameter, *b*: tumor short diameter) to construct the growth curves. After the experiment, blood was taken from the heart of the mouse and then it was euthanized; the tumor tissue was excised and weighed. The tumor inhibition rate (IR) of each group was calculated by the formula, IR = (1 − experimental group tumor weight/control group tumor weight) × 100%. A portion of tumor tissue was fixed in 4% paraformaldehyde and the remaining tissue was stored in liquid nitrogen.

### 2.12. Hematoxylin and Eosin (H&E) Staining

Hematoxylin and eosin (H&E) staining and optical microscope (TEM) analysis were used to evaluate histopathological characteristics of xenografts performed as the previous description reported [13]. Briefly, the tumor tissues were fixed with 4% paraformaldehyde, embedded in paraffin, while being fixed in 2.5% glutaraldehyde and 1% osmic acid, sectioned at 5 *μ*m, stained with hematoxylin and eosin, and observed and photographed under 10x and 40x eyepieces under an optical microscope (Olympus, Tokyo, Japan).

### 2.13. Western Blotting Analysis

Protein expression of Bcl-2, caspase-3, Cyt-C, and *p*53 was detected using western blotting. Samples were washed with PBS and the appropriate amount of cell lysates was added. The tumor tissues were shaken at 4°C for 5 min and centrifuged at 4°C for 10 min (10000 ×g). The supernatant was collected and protein was extracted. 50 *μ*g protein was conducted using SDS-PAGE and was transferred to a nitrocellulose filter (NC filter). After sealing with 5% BSA solution, polyclonal rabbit anti-mouse antibodies Bcl-2 (1 : 1000), caspase-3 (1 : 1000), Cyt-C (1 : 500), and *p*53 (1 : 1000) were added to the protein and incubated overnight. TBST was used to wash the membrane 4 times. The membrane was then incubated in goat anti-rabbit IgG secondary antibody and rat anti-mouse secondary antibody (dilution at a 1 : 2000, Cell Signaling Technology) at 37°C for 1.5 h and detected with ECL kit (Pierce, MA, USA). Protein bands were analyzed and processed using ChemiDoc type gel imaging system (Bio-Rad, CA, USA).

### 2.14. Immunohistochemistry (IHC) Staining

The expression of Bcl-2, caspase-3, Cyt-C, and *p*53 was measured with immunohistochemistry. Tumor tissue was fixed with 10% neutral formaldehyde solution, paraffin-embedded, and cut into 4 *μ*m samples. Additional sections were deparaffinized through a graded series of dimethylbenzene and ethanol; tissue sections were washed gently with phosphate buffer saline, followed by incubation overnight at 4°C in solution with the Bcl-2 (1 : 500), Cyt-C (1 : 500), *p*53 (1 : 500), and caspase-3 (1 : 300) polyclonal rabbit anti-mouse antibodies (Proteintech, Wuhan, China), respectively. After washing, the sections were incubated with goat anti-rabbit IgG antibody (ab6721, 1 : 500 in PBS) for 50 min at room temperature. The sections were then washed again, incubated with 3,3-diaminobenzidine (Solarbio, DA1010), washed, and counterstained with hematoxylin. Five visual fields were selected randomly in each section and observed with a biological imaging microscope (Olympus, Tokyo, Japan) at 400x. The average optical density (AOD) was calculated.

### 2.15. Statistical Analysis

Statistical analysis was performed on SPSS 22.0 software (Chicago, IL, USA). The results were expressed as mean ± standard deviation (SD), and one-way ANOVA was used to company the results (GraphPad Prism 5.0, GraphPad Software, USA). *p* < 0.05 indicated that there are significant differences.

## 3. Results

### 3.1. Quality Control and Identification of HXHJ

The chemical constituents of the HXHJ sample were analyzed by HPLC-ESI-QTOF-MS/MS in the positive and negative ionization modes. In the full scan mass spectra, the identified compounds are mainly exhibited in the form of [M-H]^−^. The UV and total ions chromatogram of ESI(-) are shown in Figure 1. The retention time, molecular formula, name, and source of 19 compounds are summarized in Table 1. Among them, the contents of apigetrin, apigenin, and aloe emodin were 23.6%, 9.5%, and 24.3%, respectively. To ensure the quality of the HXHJ used in this study, apigetrin, apigenin, and aloe emodin were used as quality control standards.

### 3.2. HXHJ-Medicated Serum Induced the Proliferation and Morphological Changes of HepG2 Cells In Vitro

**Figure 2:**
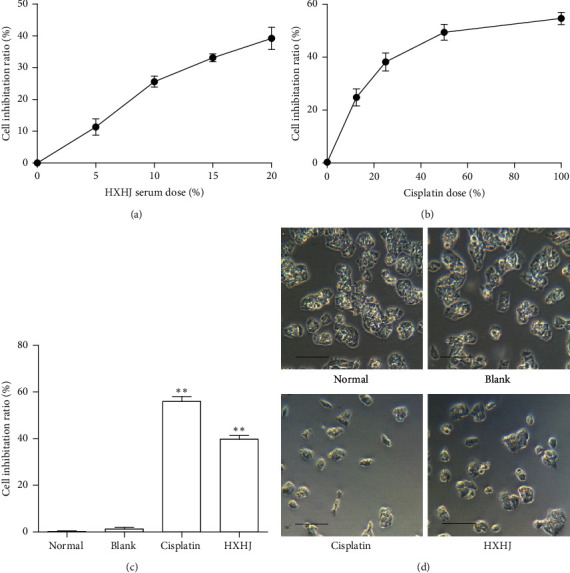
HXHJ-medicated serum induced HepG2 cell proliferation inhibition and morphological changes. (a) CCK8 determination. The cells were treated with different percentages of HXHJ-medicated serum to obtain the corresponding cell inhibition rate (mean ± SD). (b) 20% HXHJ-medicated serum inhibits the proliferation of HepG2 cells and (c) influences cell morphology under an inverted microscope. ^*∗∗*^*p* < 0.01 versus blank group. Data, mean ± SD (*n* = 3, bar = 50 *μ*m, ×100).

In order to know the cytotoxicity of HXHJ, we used the CCK8 method to detect the anti-HepG2 cell proliferation effect of HXHJ-containing serum and found that the blank serum had basically no effect on the proliferation of HepG2 cells. The 20% HXHJ-containing serum had the best anti-HepG2 cell proliferation effect. The inhibition rate was 39.15% (*p* < 0.01) (Figure 2(a)). Taking 100 *μ*mol/L cisplatin as a positive control, the effect trend of 100 *μ*mol/L cisplatin and 20% HXHJ-containing serum is the same compared with the normal group and blank group, and the changes in cell morphology were observed. It was found that, compared with the blank serum group, HXHJ-containing serum reduced cellular adhesiveness, fuzzy cellular structure, and cellular number (Figures 2(b) and 2(c)). These results suggest that HXHJ can effectively inhibit the proliferation of HepG2 cells and cause morphological changes.

### 3.3. HXHJ-Medicated Serum Induces Apoptosis of HepG2 Cells In Vitro

**Figure 3:**
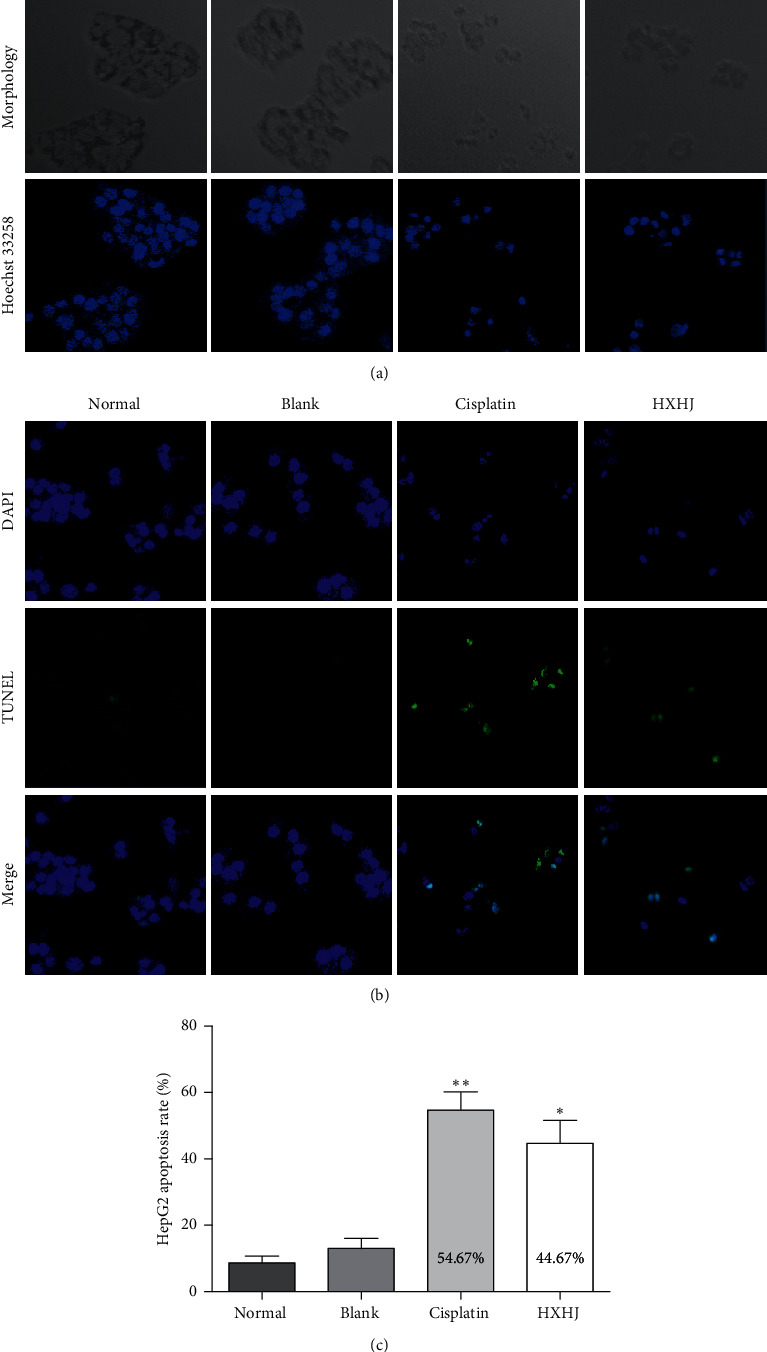
HXHJ-medicated serum induced apoptosis of HepG2 cells. (a) Hoechst staining. Observe the apoptosis of HepG2 cells by fluorescence microscope. (b) TUNEL staining. Observe the apoptotic number of HepG2 cells and count the apoptosis rate. Data, mean ± SD, *n* = 3, ×400. ^*∗*^*p* < 0.05, ^*∗∗*^*p* < 0.01, versus blank group.

In order to further evaluate the mechanism of HXHJ-medicated serum inhibiting the proliferation of HepG2 cells, we also detected cell apoptosis by Hoechst staining (Figure 3(a)) and TUNEL staining (Figure 3(b)). Compared with the blank serum group, we found that HepG2 cell nuclei appeared densely stained when they are treated with 20% HXHJ-medicated serum. The nuclei were on one side; some of them emitted strong blue-white fluorescence and even nuclear fragmentation and other typical apoptosis features. The number of TUNEL positive cells and the positive cell rate increased significantly (*p* < 0.01). So, we speculated that HXHJ-medicated serum inhibits cell proliferation mainly due to the induction of cell apoptosis.

### 3.4. HXHJ Inhibits Tumor Growth in Nude Mice with HepG2 Xenograft Tumors In Vivo

**Figure 4:**
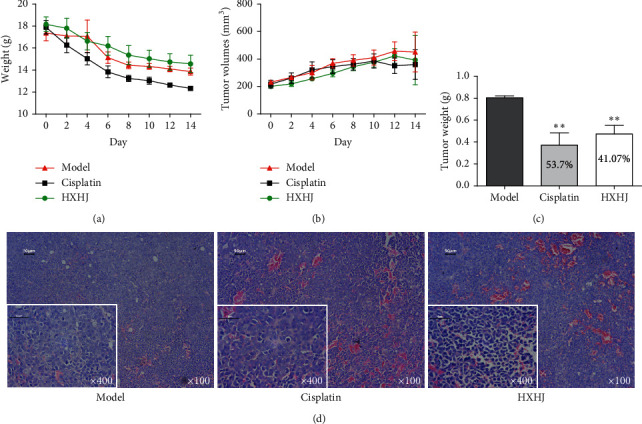
HXHJ inhibits tumor growth of HepG2 xenograft tumor in nude mice. (a) Comparison of the body mass of nude mice with HepG2 xenograft tumors in each group. (b) Tumor volume change. (c) Tumor weight and tumor inhibition rate. Data, mean ± SD, *n* = 6, ^*∗∗*^*p* < 0.01, versus model group. (d) HE staining. Observe the morphological changes of tumor tissue cells (*n* = 3, scale = 50 *μ*m, ×100, ×400).

In order to verify the antitumor effect of HXHJ in vitro and in vivo, the HepG2 xenograft tumor nude mouse model was employed to examine the effect of HXHJ in HCC. As the experimental cycle progressed, the weight of the tumor-bearing mice decreased. The weight loss of the positive drug group was the most significant. The weight loss trend of the HXHJ administration group was gentle, and the weight loss was less than that of the model group and the positive administration group (Figure 4(a)). Tumor weight and volume were significantly suppressed after HXHJ administration (*p* < 0.05), and the tumor inhibition rate was 41.07% (Figures 4(b) and 4(c)). Observed under an optical microscope, it was found that most of the cancer cells in the model group had irregular shapes, rich cytoplasm, and typical malignant tumor cell morphology, with large areas of cell-free eosinophilic staining. After HXHJ treatment, most tumor cells ruptured, the nuclei were pyknotic and deeply stained, and cell apoptosis appeared (Figure 4(d)).

### 3.5. HXHJ Regulated the Expression of Apoptosis-Related Protein Pathway *p*53/Bcl-2/Cyt-C/Caspase-3 In Vivo and In Vitro

**Figure 5:**
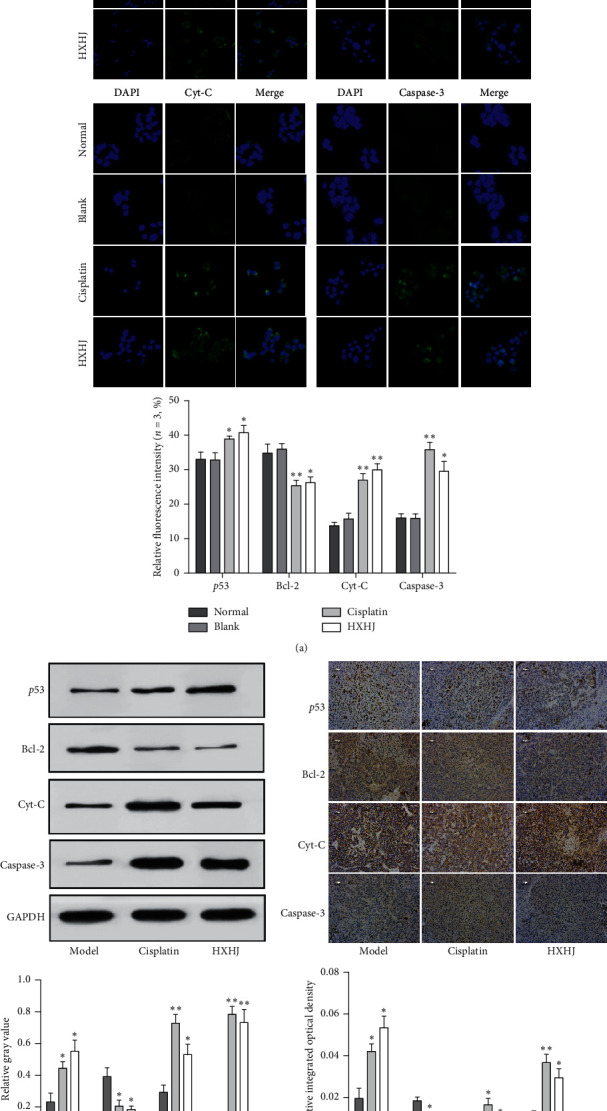
HXHJ regulated the expression of *p*53/Bcl-2/Cyt-C/caspase-3 in vivo and in vitro. (a) Immunofluorescence. Observe the protein fluorescence intensity under a laser confocal microscope and use the average cell fluorescence intensity value to reflect the relative expression level of each protein (×400). Data, mean ± SD, *n* = 3. ^*∗*^*p* < 0.05, ^*∗∗*^*p* < 0.01, versus blank. (b) Western blotting. Image J software was used to analyze and process protein gray-scale bands. (c) Immunohistochemistry. Observe and count the integral optical density value of protein under a biological imaging microscope (×400). *p*53: tumor suppressor protein. Bcl-2: B-cell lymphoma 2 gene. Cyt-C: apoptosis-related protein cytochrome C; caspase-3: aspartate proteolytic enzyme-3. Data, mean ± SD, *n* = 3. ^*∗*^*p* < 0.05, ^*∗∗*^*p* < 0.01, versus model.

To further explore the mechanism of HXHJ-induced apoptosis in hepatocellular carcinoma, we also detected the expression of apoptosis-related protein pathway *p*53/Bcl-2/Cyt-C/caspase-3. Among them, Cyt-C efflux was the core process of mitochondrial apoptosis pathway; *p*53 could downregulate the expression of antiapoptotic protein Bcl-2, induce the release of apoptosis-forming factor Cyt-C, and recruit and activate caspase-3, thereby promoting cell apoptosis. In in vitro experiments, we used immunofluorescence to detect the expression of apoptosis-related proteins. The fluorescence intensity and localization of each protein were observed under a laser confocal microscope, and it was found that, in intervention treatment with 20% HXHJ-containing serum, the relative fluorescence intensity of Bcl-2 in HepG2 cells was significantly reduced, and the relative fluorescence intensity of caspase-3, Cyt-C, and *p*53 was significantly increased (*p* < 0.01, Figure 5(a)). In in vivo experiments, both western blotting (Figure 5(b)) and immunohistochemistry (Figure 5(c)) were used to quantitatively and qualitatively detect apoptosis-related proteins. It is found that the change trend of the integral optical density value and relative protein gray value of each protein was consistent with the in vitro study. Therefore, we speculate that the mechanism by which HXHJ induces hepatocellular carcinoma apoptosis and hinders its proliferation may be related to the regulation of the expression of mitochondrial apoptosis-related protein pathway *p*53/Bcl-2/Cyt-C/caspase-3.

## 4. Discussion

Hepatocellular carcinoma (HCC) is one of the most common malignant tumors in the clinic, its incidence ranks 6^th^ and mortality ranks 2^nd^ among systemic malignancies. It is becoming the second leading cause of cancer deaths worldwide. As many as 800,000 patients died from HCC, which seriously affected the quality of human life [14]. Early HCC could be treated by local ablation of radiotherapy and chemotherapy, surgical resection, or liver transplantation. Despite the gradual progress of treatment, due to the late diagnosis of HCC, being prone to tumor recurrence and metastasis, and poor prognosis, the long-term overall survival time and outcome of HCC patients remain unchanged [15]. Apoptosis or programmed death plays a vital role in maintaining cell homeostasis and is considered to be the main mechanism of chemotherapeutic agents in eradicating cancer cells [16]. As we all know, HCC is a complex systemic disease that involves the interaction of multiple targets and signaling pathways; among them, the active antiapoptotic mechanism and metastasis in tumors may be the main reason for the failure of HCC treatment [17]. Previous studies have shown that the currently popular and effective first-line drugs, targeting kinase inhibitors sorafenib and regorafenib, could effectively induce apoptosis and inhibit metastasis of cancer cells [18, 19]. However, patients experienced hand-foot skin reactions, diarrhea, weight loss, and other adverse reactions. Therefore, there is an urgent need to better understand the potential mechanism of hepatocellular carcinoma and explore new safe and effective drugs to induce hepatocyte apoptosis.

As a unique biomedicine resource, Chinese herbal medicine has been widely used in the prevention and treatment of hepatocellular carcinoma, which can effectively relieve clinical symptoms, delay tumor progression, and improve the quality of life of patients [20]. Modern studies have shown that [21, 22] *Astragalus* has a good effect of inhibiting the growth and promoting apoptosis of cancer cells. Huanglian Jiedu decoction and its constituent herbs have a good therapeutic effect on HCC, can effectively induce cancer cell cycle arrest, and attenuate the expression of the antiapoptotic protein Bcl-x in HepG2 cells. The traditional Chinese medicine compound preparation Huxie Huaji (HXHJ) Ointment used in this study is a clinically proven prescription based on the basic pathogenesis of “deficiency, blood stasis, and toxin” in Traditional Chinese Medicine, through the combination of symptoms and precise compatibility. The prescription adjuvant clinical chemotherapy and single administration have a significant curative effect on liver cancer, and long-term use has a few side effects, but the mechanism of action is not clear.

Previous studies have found that HXHJ could significantly inhibit the proliferation of liver cancer cells H_22_, induce the imbalance of the expression ratio of Bax and Bcl-2 proteins in the cells, and promote the apoptosis of cancer cells [11]. In order to further clarify the curative effect of HXHJ against HCC, we used the CCK8 method to analyze and evaluate the viability of HepG2 cells and found that 20% of HXHJ-containing serum has a good inhibitory effect on the proliferation of HepG2 cells in in vitro studies. At the same time, through the double verification of Hoechst and TUNEL staining methods, the above-mentioned inhibition of HepG2 cell proliferation may be closely related to the induction of cell apoptosis. In vivo experiments have also proved that HXHJ could effectively inhibit the growth of xenotransplanted HepG2 cell nude mice tumors and induce tumor cell rupture and apoptosis.

Mitochondria play a central and multifunctional role in the proliferation and growth of malignant tumor cells; the reduction of membrane potential and the release of proapoptotic proteins are important links in causing cell apoptosis [23]. In previous studies, it was found that the tumor suppressor gene *p*53 in liver cancer patients was obviously lost or mutated, and cancer cell apoptosis was significantly inhibited [24, 25]. *p*53 is the most common mutated gene in human cancers, can initiate various cell responses, and can cause cell cycle arrest and apoptosis, which plays an important role in the mitochondrial apoptotic pathway, and its activation can directly induce the expression of the proapoptotic protein Bax [26]. Bax, located on the cell mitochondrial membrane, is an important member of the Bcl-2 family and the main protein that regulates cell apoptosis and has become an important drug target [27]. Under the stimulation of the apoptotic signal *p*53, Bax can form a transmembrane channel or activate mitochondrial PT pores on the outer mitochondrial membrane through oligomerization, the integrity of the membrane was reduced, the membrane potential was decreased or lost, and apoptosis was increased [28]. The antiapoptotic protein Bcl-2 hindered the flow of Ca^2+^ from the endoplasmic reticulum to the cytoplasm and combined with Bax to form a Bax-Bcl-2 heterodimer, thereby preventing the occurrence of cell apoptosis [29]. On the one hand, the imbalance of the Bax/Bcl-2 ratio formed a large number of Bax-Bax homodimers to promote the permeabilization of the mitochondrial membrane; on the other hand, it induced the release of the apoptotic factor cytochrome C (Cyt-C) [30]. A large amount of Cyt-C was compounded with caspase-9 and apoptotic enzyme activator Apaf-1 to form apoptotic bodies, which triggered the self-activation of caspase 9, then activated the apoptotic executive protein caspase-3 and caspase-dependent nucleases, and promoted the increase of cell apoptosis [31, 32].

In order to further explore the mechanism of HXHJ inducing apoptosis of HCC cells, our study combined in vivo and in vitro experiments; used immunofluorescence, western blot, and other methods; and found that HXHJ may regulate the expression of tumor cell signaling pathway *p*53/Bcl-2/Cyt-C/caspase-3, which induced mitochondrial cell apoptosis, thereby exerting a good anti-HCC effect. 3-Kinase/protein kinase B (PI3K/Akt) and mitogen-activated protein kinases (MAPKs) are also regulated by mitochondrial signaling [33]. In addition, the apoptotic pathway is not only related to the mitochondrial apoptotic pathway but also related to the death receptor pathway, mainly by recruiting the Fas-related death domain (FADD) adaptor protein and activating the procaspase-2 precursor to start the cell apoptosis [34]. Chinese herbal medicine has unique multipathway and multitarget regulation characteristics. Whether the anti-HCC effect of our HXHJ is also related to the regulation of the above-mentioned signal pathways needs to be further explored.

## 5. Conclusions

HXHJ Ointment had a significant effect on promoting apoptosis of tumor cells. The mechanism may be related to activating the *p*53/Bcl-2/Cyt-C/caspase-3 signaling pathway and inducing mitochondrial cell apoptosis.

## Figures and Tables

**Table 1:** Chemical components identified in the compound.

No.	tR	MS	Formula	Name	Source
1	4.108	117.0182 [M-H]^−^	C_4_H_6_O_4_	Succinic acid	CR
2	6.942	179.0164 [M-H]^−^	C_9_H_8_O_4_	Caffeic acid	CR, AR, HH, AP, SC
3	7.237	353.0888 [M-H]^−^	C_16_H_18_O_9_	3-Caffeoylquinic acid	HH, SC
4	7.284	353.0886 [M-H]^−^	C_16_H_18_O_9_	Chlorogenic acid	HH, SC, SB
5	8.425	433.0949 [M-H]^−^	C_20_H_18_O_11_	Quercetin3-*O*-*α*-*L*-arabinopyranside	HH
6	12.026	685.2349 [M-H]^−^	C_31_H_42_O_17_	Nuezhenide	FL
7	12.342	435.1227 [M-H]^−^	C_20_H_20_O_11_	Taxifolin 3-*O*-*β*-D-xylopyranoside	AP
8	13.609	447.0847 [M-H]^−^	C_21_H_20_O_11_	Quercetin	HH
9	13.942	445.0683 [M-H]^−^	C_22_H_22_O_10_	Calycosin-7-O-*β*-D-glucoside	AR
10	19.110	407.1319 [M-H]^−^	C_20_H_24_O_9_	Torachryson-8-O-glucoside	RJ
11	20.493	431.0951 [M-H]^−^	C_21_H_20_O_10_	Apigetrin	AR
12	22.510	285.0378 [M-H]^−^	C_15_H_10_O_6_	Luteolin	SB
13	27.361	283.0594 [M-H]^−^	C_15_H_8_O_6_	Dihydrokaempferol	SC
14	30.545	269.0442 [M-H]^−^	C_15_H_10_O_5_	Apigenin	SB
15	43.063	253.1067 [M-H]^−^	C_16_H_14_O_3_	6-Methoxyflavanone	HH
16	46.180	237.0540 [M-H]^−^	C_15_H_10_O_3_	2-Methyl-3-hydroxyanthraquinone	HH
17	52.097	293.2107 [M-H]^−^	C_18_H_30_O_3_	9,10-Epoxy-10,12-octadecadienoic acid	CR
18	53.514	269.0444 [M-H]^−^	C_15_H_10_O_5_	Aloe emodin	RJ
19	55.481	295.2260 [M-H]^−^	C_18_H_32_O_3_	9,10-Epoxy-12-octadecenoic acid	CR

SB: *Scutellaria barbata* D. Don; RJ: *Reynoutria japonica* Houtt; SC: *Smilax china* L.; FL: *Fructus Ligustri Lucidi*; CR: *Codonopsis Radix*; AR: *Astragali Radix*; HH: Herba *Hedyotidis Diffusae*; AP: *Agrimonia pilosa* Ledeb.

## Data Availability

The data used to support the findings of this study are included within the article.

## Abbreviations

HXHJ: Huxie Huaji Ointment
HCC: Hepatocellular carcinoma
Bcl-2: B-cell lymphoma 2 gene
Cyt-C: Cytochrome C
H&E: Hematoxylin and eosin
TCM: Traditional Chinese medicine
TCMF: Traditional Chinese medicine formulas.
